# The in-utero experience of piglets born from sows with lameness shapes their life trajectory

**DOI:** 10.1038/s41598-021-92507-2

**Published:** 2021-06-22

**Authors:** Marisol Parada Sarmiento, Thiago Bernardino, Patricia Tatemoto, Gina Polo, Adroaldo José Zanella

**Affiliations:** 1 Center for Comparative Studies in Sustainability, Health and Welfare, Department of Preventive Veterinary Medicine and Animal Health, School of Veterinary Medicine and Animal Science, University of São Paulo, Campus Fernando Costa, Av. Duque de Caxias Norte, 225 Caixa Postal 23, Pirassununga, SP CEP 13635-900 Brazil; 2 Faculty of Veterinary Medicine, University of Teramo, Piano d’Accio, 64100 Teramo, Italy; 3 Grupo de Investigación en Epidemiología y Salud Pública, Universidad de La Salle, Bogotá, Colombia

**Keywords:** Social behaviour, Stress and resilience, Animal behaviour

## Abstract

Experiences during gestation can alter the mother’s behavior and physiology, thereby potentially affecting the behavioral and physiological development of the offspring. In livestock, one common challenge for pregnant animals is lameness: a multifactorial condition that causes pain, stress, resulting in poor welfare outcomes. Since maternal pain can affect offspring development, we aimed to quantify the behavioral response in 142 piglets born from sows with different degrees of lameness during pregnancy. Gait scores of 22 pregnant group-housed sows were assessed six times at 2-week intervals. Lameness scores varied from 0 (no lameness) to 5 (most severe lameness score). Saliva samples and behavior were assessed in the sows throughout pregnancy. Sows were moved to individual farrowing pens and placental tissue was collected for glucocorticoid assessment. At 28 days of age, piglets were weaned, weighed, and regrouped by body size and sex. Skin lesions were counted for each piglet on days 28, 29, and 30 after birth. During open field and novel object tests on day 30, the vocalization and activity levels were evaluated. Piglet data were grouped by the lameness score of the sows as G1 (without lameness), G2 (moderate lameness), and G3 (severe lameness). Data analysis included ANOVA or Kruskal–Wallis tests and pairwise comparisons which were performed using Tukey and Kramer (Nemenyi) test with Tukey-Dist approximation for independent samples. G2 piglets were heavier than G3 at weaning. G1 piglets had fewer skin lesions at days 28 and 29 than G2 piglets. Moreover, G1 piglets vocalized more than G2 when they were subjected to the combined open field and novel object test. We did not identify differences among sows showing different lameness scores in the concentration of placental or salivary glucocorticoids. Lameness in pregnant sows altered the offspring’s weight gain, number of skin lesions and vocalizations, together showing evidence that lameness in sows affect offspring performance and behavior.

## Introduction

Lameness in pregnant sows is a common and painful condition and is one of the most frequent reasons for culling, causing considerable economic losses^1,2^. It is also recognized as a very important indicator of animal welfare^3^. Lameness can be the consequence of several factors including inadequate handling, improper housing conditions, and deficient nutrition. High sow density and poor flooring conditions can trigger lameness which can be exacerbated by post-mixing aggression^4^. Furthermore, nutritional factors such as mineral and vitamin deficiencies may be detrimental to bones, articular cartilages, and claws^5^. Lameness can cause health, behavioral, and physiological alterations in animals. The main health impairments associated with lameness in sows include traumas, fractures, osteochondrosis, and foot lesions^1^. Behavioral modifications involve a decrease in social interactions, exploratory behavior^6^, and alterations in feeding and lying behavior^7,8^.

The behavioral changes associated with lameness indicate that the condition causes pain^6,9,10^. Objective assessment of lameness is established using scoring systems based on behavioral changes, caused by pain, that can distinguish levels of severity^11^. According to the International Association for the Study of Pain (IASP), pain can be defined as an unpleasant sensory and emotional experience associated with actual or potential tissue damage^12^. Physiological responses to painful stimuli can be measured by changes, which are mostly mediated by the sympathetic nervous system and by the hypothalamic–pituitary–adrenal axis (HPA). Measurement of catecholamines and changes in autonomic responses, such as respiratory rate, heart rate, blood pressure and body temperature, are indicators of sympathetic system activation. Measures usually used to assess HPA responses, such as production of glucocorticoids^13^ are reported in animals experiencing pain. Painful stimuli also leave traces at the molecular level, altering inflammatory markers such as TNFα, C-reactive protein, and several interleukins^14^. Since pain is challenging and can be stressful, this scenario can worsen welfare outcomes in pregnant animals due to physiological and molecular responses that could result in epigenetic changes affecting offspring developmental outcomes. Studies have shown that stress or inflammatory responses during pregnancy alters the development of brain structures in the offspring, mainly those pathways responsible for memory, social behavior and emotions^15,16^.

Glucocorticoids play a fundamental role during pregnancy in the normal development of fetal organs. Glucocorticoid increase as a result of adverse situations in pregnant subjects can impact fetal development affecting mainly the hippocampus, HPA axis functions and behavior^16,17^. The main placental protective system against active and high glucocorticoid levels is the placental enzyme 11 beta-hydroxysteroid dehydrogenase (11β-HSD-2), responsible for the inactivation of cortisol by conversion to cortisone. A failure in this system has negative consequences in fetal programming^18^.

It has been reported that a balance between embryotrophic and embryotoxic cytokines in the female reproductive tract is determined by several stressful events, impacting embryo implantation, placental development, and fetal growth. These mediate biological effects of embryo programming, embryo plasticity, and adaptation^19,20^. In a gene expression study using mononuclear cells from cows with and without lameness an up-regulation of the GM-CSF-R-alpha gene in lame relative to sound cows was reported^21^. In another study the cytokine GM-CSF was identified as a physiological regulator of fetal growth trajectory and placental morphogenesis^22^.

Painful conditions during pregnancy are sources of prenatal stress, which involve a cascade of physiological and molecular responses with potential to reprogram epigenetically genes involved in the development of stress neurocircuitry in the offspring, producing phenotypic modifications such as increased basal glucocorticoid levels, decreased expression of glucocorticoid receptors in the hippocampus and changes in spatial learning or in memory performance^23^.

Lameness is a painful and stressful condition for the sow which has the potential to affect fetal development, the changes being mediated by high glucocorticoid concentration, cytokines and other stress biomarkers that could cause epigenetic changes.

Given the impact of maternal pain in modulating coping systems in the offspring brain^24^, we aimed to measure developmental outcomes in piglets born from sound and lame sows. We hypothesized that offspring born from sows with lameness during pregnancy would show changes in fear responses to novel situations, and also in aggressive behavior, which can, in turn, affect performance outcomes. Specifically, we hypothesized that during an open field and novel object test, piglets from lame sows would explore the arena less and would show longer latency to explore a novel object when compared with offspring of non-lame sows. Furthermore, we hypothesized that piglets from sows without lameness would cope better with social conflicts, thus having fewer skin lesions when compared with piglets born from lame sows.

## Materials and methods

### Ethical approval

Data were collected from the experimental pig farm of the University of São Paulo (USP), located at the Campus Fernando Costa—Pirassununga, Brazil, with the approval of the Ethics Committee on the Use of Animals (CEUA) of the School of Veterinary and Animal Science (FMVZ/USP), with the number N° 3606300114, according to the Law 11.794, of October 8, 2008 and Decree 6899 of July 15, 2009 with the rules issued by the National Council for Control of Animal Experimentation (CONCEA)—Brazil. The study was carried out according to the ARRIVE guidelines (https://arriveguidelines.org/). The approval of the ethics committee is placed as Supplementary Information.

### Animals, facilities, and handling

The present data were collected during a concomitant experiment which measured the impact of fiber in the diet of gestating sows on their offspring^25^.

Data from 22 pregnant sows (F1 Landrace × Large White) and their offspring were studied. The sows were nulliparous and healthy at selection, and subsequently inseminated in the same period of the year with pooled semen from a specified group of boars. After insemination and until day 107 of gestation, sows were housed groups of nine in pens that measured 6.7 m × 4.4 m (3.3 m^2^ per sow). Each group was fed in nine individual feeding stalls (1.8 m × 0.55 m) two times per day—morning and afternoon—and water was supplied *ad libitum* through nipple drinkers. Individual food consumption was measured only during gestation, not during lactation.

On day 107 of gestation, the sows were moved from the group pens to individual farrowing pens measuring 4.3 m × 2 m. Connected to the pen, there was a creep-feeding area made of concrete (0.97 m × 2.2 m), where piglets had unlimited access to solid feed from birth. We kept all animals in the farrowing pens until day 28 of lactation. Bedding material was provided for the sows and piglets, composed of dehydrated sugarcane bagasse and hay. Farrowing was monitored with IP video cameras (FOSCAM, Fi9821p HD 720P), with a real-time internet transmission to the experimenters. Farrowing was followed through computers, smartphones, and direct observation. All sows were fed an identical solid lactation diet with *ad libitum* access.

All piglets were weighed on the 1st, 21st, and 27th days of age. In addition, during the first day of life, routine management tasks of the farm were carried out: teeth grinding, administration of iron dextran (100 mg, intramuscular), and identification by an ear notch under local anesthesia with 5% lidocaine cream. Weaning occurred at 28 days of age and the piglets were moved to experimental pens (1 m × 0.75 m) with slatted plastic floors. Weaned piglets were mixed into groups formed from two different litters, allocating four piglets matched by weight and sex per pen (for more details see Bernardino et al.^25^). Food and water were provided *ad libitum*, and the pen was cleaned daily.

### Experimental design

To assess the effects on the offspring of lameness during gestation, we assessed lameness scoring 22 sows, six times throughout gestation^26,27^. The behaviors of sows, salivary, and placental glucocorticoid concentration were analyzed^28^. In the offspring (N = 156 piglets), aggressiveness was assessed indirectly by counting skin lesions^25,29^ and behavior was assessed with a combination of open field and novel object tests^30^. An explanation of the experimental design can be seen in Supplementary Fig. S1.

### Lameness assessment in sows

During gestation, six lameness assessments were performed with intervals of 2 weeks between measures. The lameness score applied was a combination of two validated score systems (Table 1)^26,27^.

**Table 1:** Locomotion score system to assess gait in sows . adapted from Refs.^26,27^.

Degree of lameness	Description
0	The animal moves easily with little stimulation and bears weight comfortably of all its legs
1	Minor alterations in the gait. When standing, the sow alternates weight bearing in legs. It still walks easily
2	Locomotor disturbance is perceptible in the gait, shorting the steps. Alters position and support of the legs when standing
3	Supports the limb with difficulty. Shortened stride. Reluctant to bear weight on the affected limb
4	Lameness of one or more limbs, display of compensatory behaviors such as arching of the back and/or squatting of the head. Reluctance to walk, difficult to move from one place to another
5	Try to lie down, get up with difficulty and try not to support the committed leg(s)

According to the lameness score, sows were classified into three groups, G1: Sows with a degree of lameness ≤ 1 in all six lameness assessments. G2: Sows with a degree of lameness ≤ 3, with at least one of the six lameness assessments with degree 2 or 3. G3: Sows with a degree of lameness ≤ 5, with at least one of the six lameness assessments with degree 4 or 5.

When lameness was detected, pain treatment was performed according to a standard procedure; Flunixin Meglumine at 2.2 mg/kg was administrated intramuscularly, once a day, for 4 days to sows with locomotion score ≥ 3.

### Pregnancy and farrowing

During pregnancy behaviors related to position and activity were collected on days 29, 30, 31, 59, 60, 61, 74, 75, 76, 89, 90, and 91, in four periods per day: before and after feeding in the morning, and before and after feeding in the afternoon. During each period, each animal was observed three times, each lasting 2 min, for a total of 6 min per period and 24 min per day of observation. The behaviors observed were sleeping, lying ventrally, lying laterally, standing, sham-chewing, rooting the floor, rooting on the empty feeder, licking the floor, interacting with mats, and interacting with fences or gates. The details of each behavior are in Table 2.

**Table 2:** Sows behaviors collected on first, second and last third of gestation, before and after feeding. . Adapted from Ref.^31^.

Behavior	Definition
Sleeping	Sleeping animal
Lying ventrally	Lying with belly facing the ground with all limbs under the body
Lying laterally	Lying sideways, with all the limbs extended laterally
Standing	Body supported by the four limbs
Shame-chewing	Continuous chewing without the presence of visible food in the oral cavity
Rooting the floor	Snout touches the ground followed by head movements
Rooting on the empty feeder	Snout touches the empty feeder followed by head movements
Licking the floor	The tongue touches the floor and is followed by movements with the head
Interacting with mats	Snout or tongue touches mats followed by head movements
Interacting with fences or gate	Biting or nibbling the fence wire or gate

Saliva samples were collected on the same days that behavioral assessment was carried out, early in the morning (06:00) and in the late afternoon (18:00). These samples were stored at – 20 °C immediately after collection and cortisol was measured with Enzyme Immunoassay (EIA)^32^. The collection methodology used was adapted from Refs.^33,34^, using two hydrophilic cotton rolls tied to a dental floss with long tips and presented to each animal.

After farrowing, four placenta samples of the same size were collected from random locations from 19 sows and stored immediately at – 20 °C. Glucocorticoid extraction was performed to measure cortisol and cortisone levels using an EIA^32^. The placentas of three sows were not collected due to unforeseen problems.

Additional data collected, included pregnancy length, the total number of piglets born, total piglets born alive, total litter weight at farrowing, total litter weight at 21 and 27 days of age, average daily litter weight gain, average daily weight gain per animal and number of crushed piglets.

### Post-mixing aggression score

Data from 156 piglets from the 22 sows were collected and analyzed according to the score of lameness measured in the sow. The number and distribution of the individuals in each group are shown in Table 3.

**Table 3:** The number (n) of sows and piglets studied per group.

Group	Degree of lameness	Number of sows	Number of piglets
G1	0–1	7	52
G2	2–3	10	66
G3	4–5	5	38
Total	–	22	156

**Figure 1:**
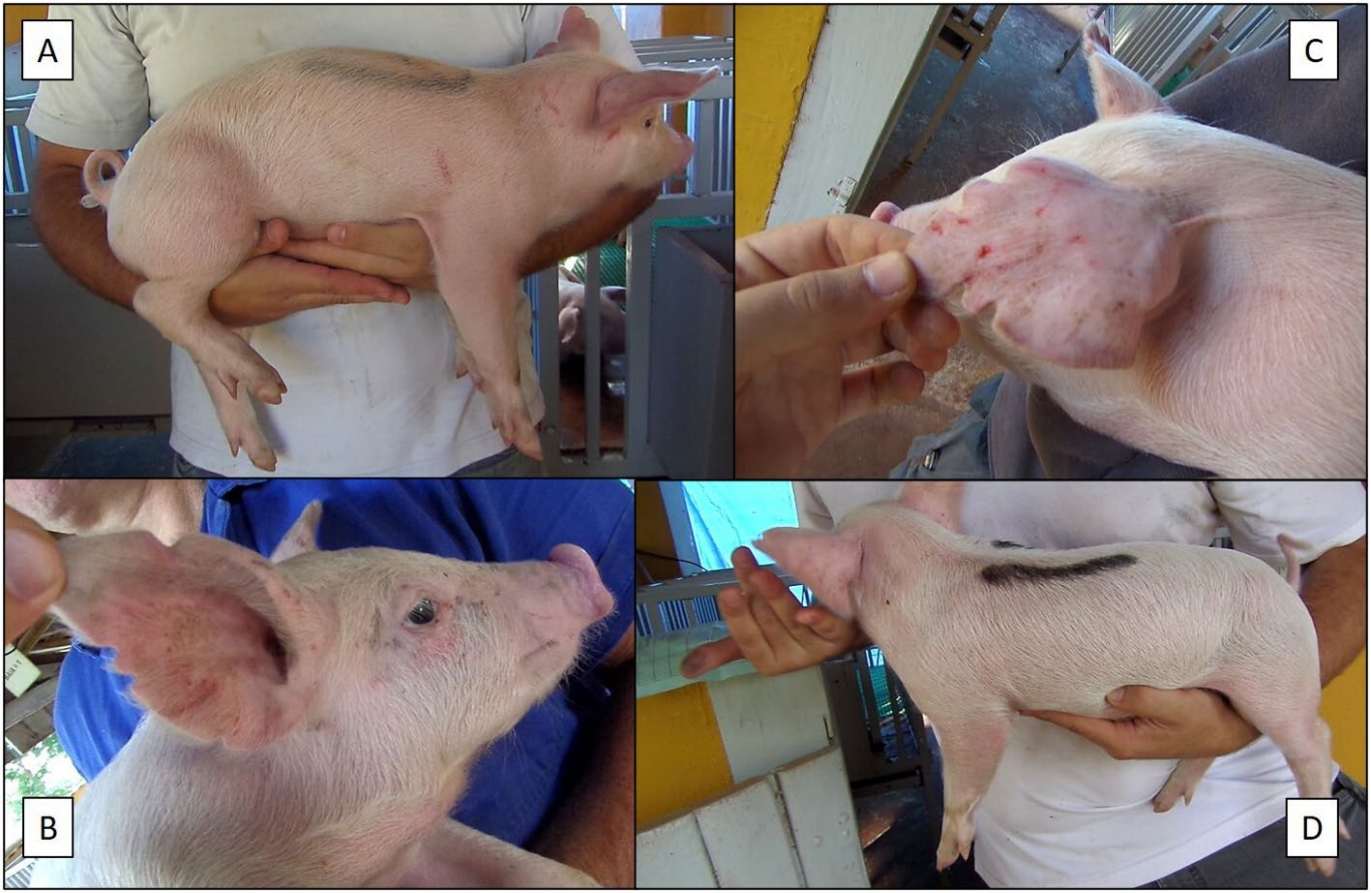
Examples of images used to count skin lesions. (**A**) Right lateral body; (**B**) face and right lateral ear; (**C**) back of the left ear and (**D**) left lateral body.

Pre- and post-mixing aggression was assessed, based on validated methodology^25,29^. Photographs and videos of each piglet were taken daily at 28, 29, and 30 days of age (see Fig. 1). Six piglets from each sow (three males and three females) were used for the evaluation of skin lesions. Two independent evaluators, blind to the treatments, counted skin lesions using the photographs and videos.

### Open field and novel object test

**Figure 2:**
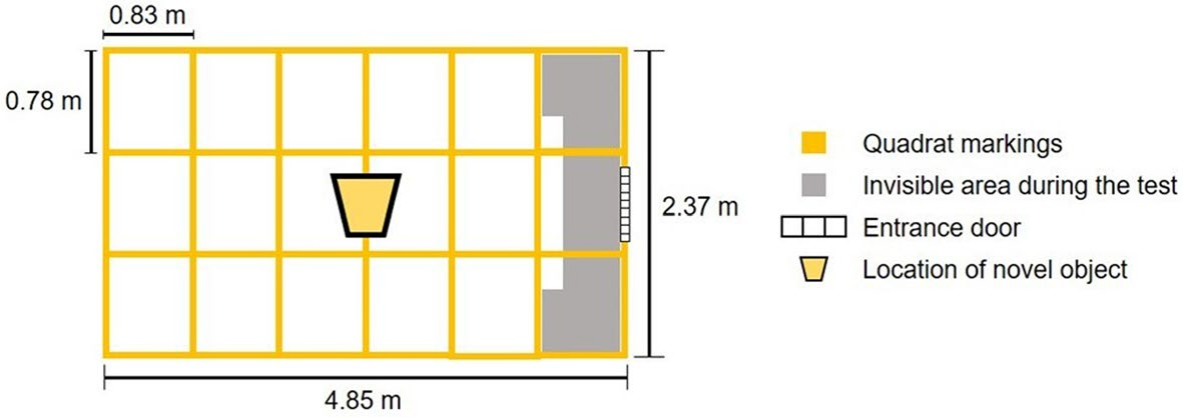
Graphic representation of pen used to perform the open field and novel object tests.

A combination of open field and novel object tests were performed on 142 piglets at the end of the experiment, when pigs were 30 days of age using the methodology previously described^30^ to assess activity levels, exploratory behavior, and vocalizations (Table 4). The tests were carried out in a pen with the floor marked with squares (see Fig. 2). To carry out the tests, the piglets were taken randomly, alternating between males and females. Due to unforeseen difficulties, the tests could not be done on all piglets; only 142, out of the 156 piglets, were assessed in both tests.

**Table 4:** Description of data collected during open field and novel object test.

Test	Measure	Description
Open field test	Latency	Time in seconds between piglet entering in the pen and walking
	Activity	Time in seconds spent walking
	Quadrants accessed	Time in seconds spent in central and lateral quadrants (quadrants on the edge of the pen)
	Vocalizations	A count of all types of vocalization
Novel object test	Latency	Time in seconds between the bucket being placed in the pen until animal interaction with the object (close to and with the head toward to the object)
	Near to the object	Time in seconds the animal spent close to the object (in quadrants that surround the object)
	Quadrants accessed	Time in seconds spent in central and lateral quadrants (quadrants on the edge of the pen)
	Vocalizations	Number of all types of vocalizations

The open-field test consisted of positioning each piglet at the same starting point in the pen, in order to assess the time taken to move, time spent walking, and time remaining in peripheral or central squares. Immediately following the open field test, the novel object test was carried out, and involved introducing an unknown object into the pen to assess latency, exploratory behavior, and proximity to the object. A yellow empty polypropylene bucket with a capacity of 20 L was used as an unknown object. To avoid visual contact between the piglets and the experimenter, a pulley mechanism was used to introduce the bucket in the pen. In both tests, all types of vocalization were counted. For each individual the tests lasted 10 min: 5 min for the open field test followed by 5 min for the novel object test. To reduce the possible chemical signals present in the environment, the pen was always washed with water prior to each piglet assessment.

Open field and novel object tests were recorded with a digital camera (Samsung WB250F Smart Wi-Fi Digital).

### Statistical analysis

Multiple correspondence analysis (MCA), a type of multivariate analysis, was employed to construct relationships among piglets variables (weight at birth, skin lesions, latency/vocalizations in the open field test, latency/vocalizations/exploration in the novel object test) or sows variables (born piglets, alive born piglets, average weight at birth, average daily weight gain, sow weight, salivary cortisol ratio at pregnancy, placental cortisone and cortisol concentration), the presence of high/low fiber diet of sows (used in the first experiment^25^) and its association with the different lameness groups. The associations were confirmed using a Chi-square test.

To determine the residual distribution was used Shapiro–Wilk test with all variables, when the result was < 0.05 a non-parametric test was used, and when it was > 0.05 a parametric test was used, always considering the number of groups to be compared. The variables used to perform a Shapiro Wilk test were in sows: performance data, ratio between morning/afternoon saliva cortisol concentration and placental cortisol/cortisone concentrations; in piglets: skin lesions number, weight at birth, 21 and 27 days of age, open field, and novel object tests data.

To analyze saliva cortisol at 75 and 90 days of pregnancy, we calculated the ratio between morning and afternoon of each day, ratio was calculated dividing morning cortisol concentration into afternoon cortisol concentration. Subsequently, a Kruskal–Wallis test was used to compare the ratio between groups. Placental cortisol and cortisone were examined in an intraspecific and interspecific way.

Intraspecifically, a T-test was performed, comparing placental cortisol with placental cortisone from the same group. Interspecifically a One-way ANOVA was performed, comparing placental cortisol or placental cortisone from different groups. We calculated the ratio between placental cortisol and cortisone from each sow, dividing cortisol concentration into cortisone concentration and subsequently a One-way ANOVA was performed to compare it between groups.

Weight and skin lesions were compared independently for each day between groups using Kruskal–Wallis test or ANOVA.

A significance level of 5% was considered. All analyses and graphs were performed using the free software environment for statistical computing R (R version 4.0.5)^35^. The MCA was performed using the R packages "FactoMineR"^36^ and "factoextra"^37^. To calculate Pairwise Multiple Comparisons of Mean Rank Sums Extended was used the package "PMCMRplus"^38^.

## Results

After performing an MCA and confirming it with a Chi-square, no associations were found between previous nutritional treatments in sows and the results of the current study (Chi-squared test, p-value = 0.31; see Supplementary Figs. S2, S3 for details).

### Data from sows and offspring before weaning

Descriptive measures of saliva cortisol, placental cortisol/cortisone, and performance data can be found in Supplementary Tables S1, S2, and S3, respectively.

On day 75 and 90 of pregnancy, we did not find differences between lame and non-lame sows when comparing salivary cortisol ratios (Kruskal–Wallis test; p > 0.05).

No difference was found in placental cortisol and cortisone concentration between G1, G2, or G3 (One-way ANOVA test; p = 0.681 for placental cortisol, and p = 0.457 for placental cortisone), and when comparing placental cortisol with placental cortisone in each group, concentration of cortisone was always higher than cortisol (T-test; p-value ≤ 0.05; see Supplementary Table S2 for details). We did not find differences when comparing placental cortisol/cortisone ratios between sow groups (One-way ANOVA; p > 0.05). The results of performance data showed no differences when comparison was carried out between groups G1, G2, and G3 (p > 0.05).

### Body weight and skin lesions in piglets

**Figure 3:**
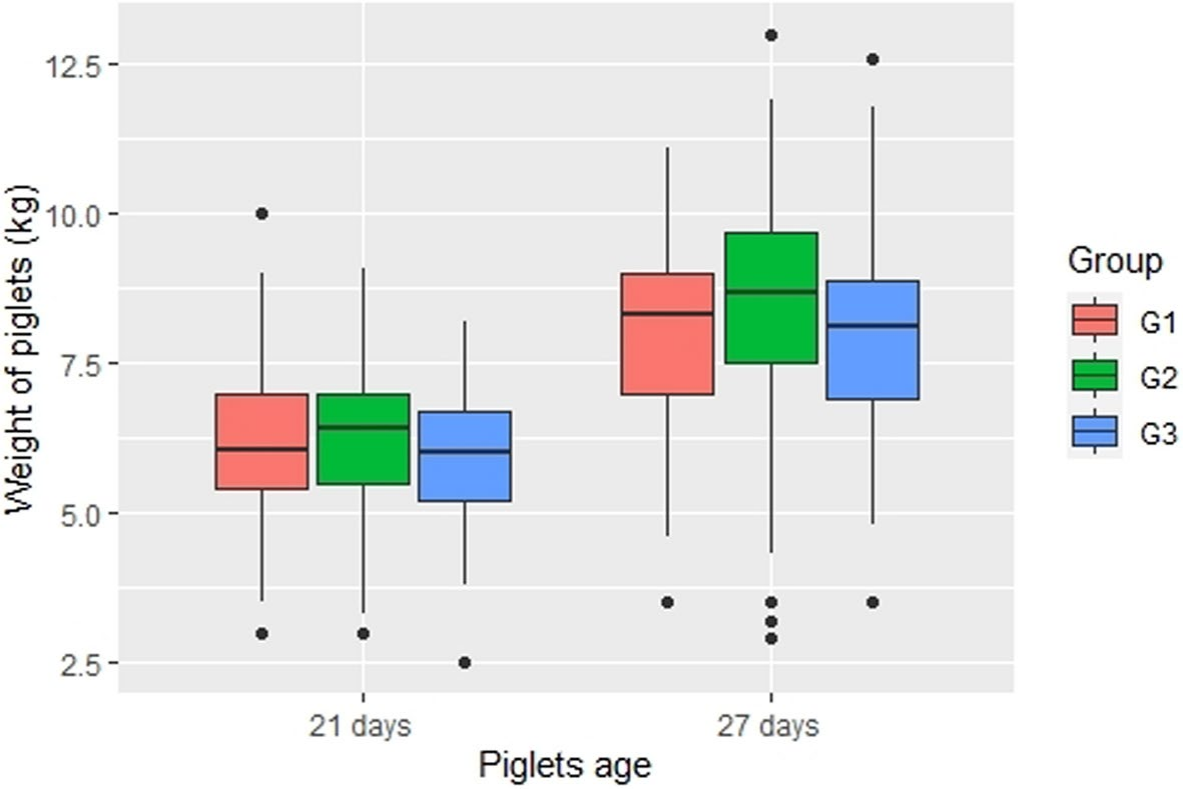
Boxplots to represent the weight of individual piglets at 21 and 27 days of age divided in three groups, according to sow lameness score (G1: lameness score 0–1; G2: lameness score 2–3; G3: lameness score 4–5). This figure was performed in the programming language R using the package ggplot2^35^.

We did not identify any weight difference at birth or at 21 days of age between piglets from groups G1, G2, or G3. However, weight at 27 days old was different between the groups (Kruskal–Wallis test; p = 0.02), with G3 piglets being lighter than G2 piglets (see Table 5 for details). A box plot of the weight of the piglets at 21 and 27 days old can be found in Fig. 3 (see Table 5 for details).

**Table 5:** Significant results of weight, number of skin lesions and number of vocalizations during open field and novel object test. The post-hoc test used to ANOVA One way was a Tukey and for Kruskal–Wallis was a Nemenyi test. Degrees of freedom always were 2.

Variable	Age days	Test	Mean			p-value	Post-hoc p-value		
			G1	G2	G3		G1–G2	G1–G3	G2–G3
Weight (kg)	27	Kruskal–Wallis	8.00	8.45	7.95	0.022	0.08	0.93	0.04
Skin lesions	28		2.69	5.08	2.95	0.003	0.01	0.99	0.02
	29		24.79	32.49	30.58	0.026	0.02	0.26	0.70
Open field test vocalizations	30	ANOVA One way	219	170	183	0.044	0.04	0.35	1.00
Novel object test vocalizations		Kruskal–Wallis	221	160	178	0.002	0.001	0.12	0.51

**Figure 4:**
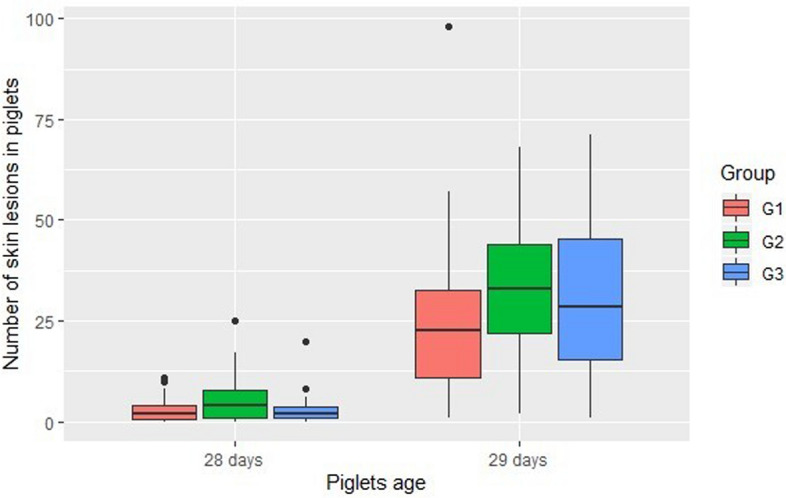
Number of skin lesions in piglets with 28 and 29 days of age, divided in three groups, according to sow lameness score (G1: lameness score 0–1; G2: lameness score 2–3; G3: lameness score 4–5). This figure was performed in the programming language R using the package ggplot2^35^.

Regarding the number of skin lesions, we found differences at day 28 and 29 (Kruskal–Wallis test; p < 0.05; see Table 5 for details). The Fig. 4 is a box plot of the number of skin lesions of the piglets at 28 and 29 days of age. On day 28, piglets from group G1 had fewer skin lesions than piglets from G2, and piglets from G3 had fewer skin lesions than piglets from G2 (see Table 5 for details). Additionally, we did not find differences between piglets from group G1 and G3. On day 29 after farrowing, we identified fewer skin lesions in piglets from group G1 when compared with piglets from G2 and no difference in the remaining comparisons (see Table 5 for details). On day 30, no difference was found between groups.

Descriptions of weight and skin lesion measures can be found in Supplementary Table S4.

### Open field and novel object test

**Figure 5:**
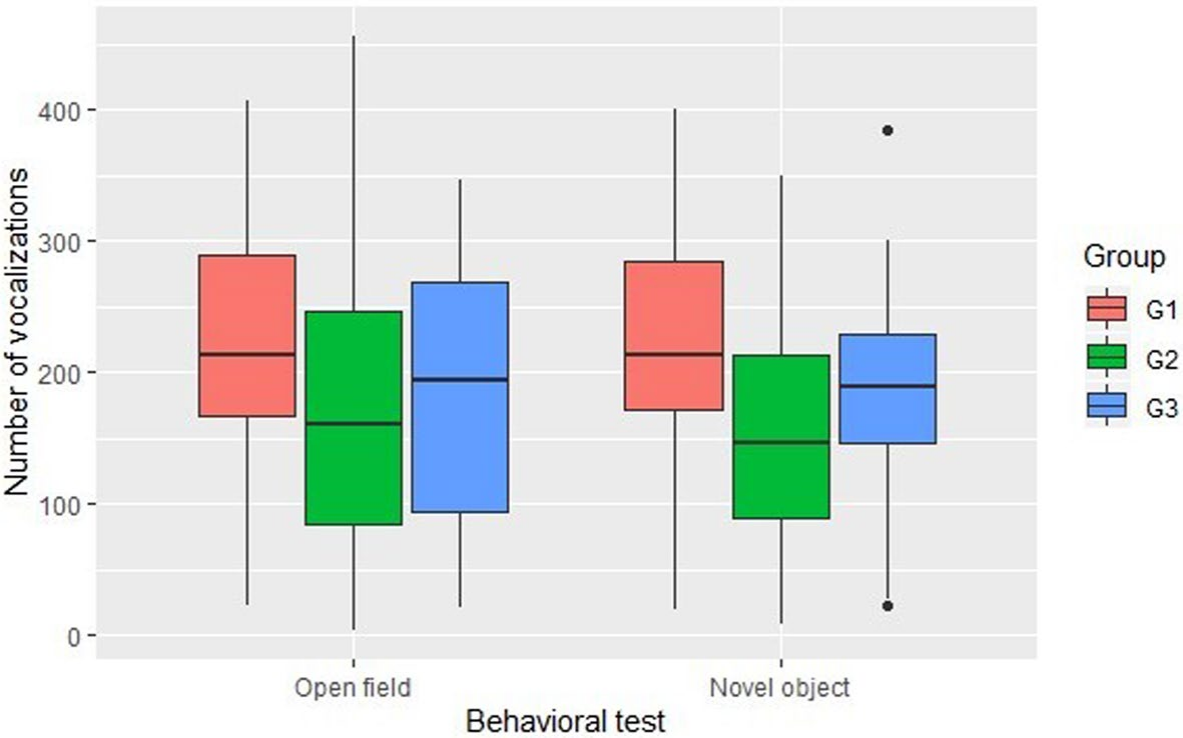
Number of piglet vocalizations during the open field and novel object test, divided in three groups, according to sow lameness score (G1: lameness score 0–1; G2: lameness score 2–3; G3: lameness score 4–5). This figure was performed in the programming language R using the package ggplot2^35^.

In the open field test, we did not identify differences in latency (Kruskal–Wallis test; p = 0.751), activity (One-way ANOVA test; p = 0.823), access to peripheral (Kruskal–Wallis test; p = 0.931) or central quadrants (One-way ANOVA test; p = 0.374). However, the number of vocalizations was higher in piglets from group G1 (offspring of non-lame sows) compared with G2 (One-way ANOVA test; p < 0.05; see Table 5 and Fig. 5 for details), G3 was positioned between them without differing from either G1 nor G3.

Similar to the results of the open field test, we found that only the number of vocalizations differed in the novel object test, with a higher number of vocalizations recorded in piglets from group G1 compared with G2 (Kruskal–Wallis test; p < 0.005; see Table 5 and Fig. 5 for details), while G3 had fewer vocalizations than G1 but more than G2, without significant differences. We did not see a difference in latency (Kruskal–Wallis test; p = 0.884), object exploration (Kruskal–Wallis test; p = 0.641), or proximity to the novel object (Kruskal–Wallis test; p = 0.254).

Descriptive measures from the variables analyzed during open field and novel object test can be found in Supplementary Table S5.

## Discussion

In this study, we have tested the hypothesis that the offspring born from sows with lameness, a painful condition experienced during pregnancy, are affected by their in-utero or neonatal experience. Here we showed that lameness, especially moderate lameness, in pregnant sows has effects on their offspring, increasing number of skin lesions, affecting weight at weaning and vocalizations during open field and novel object tests. The inconsistency of the responses observed, which did not show graded effects with the severity of the lameness, could be associated with the treatment option that our ethical review board requested, and we promptly applied to the affected animals. The use of flunixin meglumine as an analgesic treatment could potentially have mitigated the negative impact of pain on severely lame sows. Even considering the impact of the therapeutic interventions on the study, we could not let severely lame animals without treatment, for ethical reasons. The administration of flunixin meglumine, in healthy pregnant sows, as a potential control, would be of limited use, given the complex nature of the inflammatory processes associated with lameness.

Surprisingly, there were no differences in glucocorticoid concentrations in saliva or placenta among the groups indicating that other mechanisms may be associated with the reported changes in the offspring.

It has already been reported in several species that an inadequate function of the enzyme 11β-HSD-2 or high glucocorticoid concentrations during pregnancy could decrease the weight and size of the offspring^39–42^. According to the results obtained from the analysis of cortisol and cortisone concentration in the placenta, the absence of difference between the sow groups (G1, G2, and G3) showed no evidence of the role of glucocorticoid-mediated maternal stress. We anticipate that sows had effective action of the enzyme 11β-HSD-2^18^, or as the salivary cortisol data demonstrated, glucocorticoids were not the main mechanism responsible for the behavioral changes reported in this study. These findings suggests that other mechanisms could be responsible for the changes found in the offspring of sows with lameness. This could be mediated by other mechanisms associated with longer term stress that have the potential to alter fetal development^43^, or the levels of embryo toxic cytokines present in inflammation events during pregnancy^19^.

We propose that since lameness is a chronic condition^44^, and it could affect inflammatory biomarkers^45,46^, they should be evaluated in future studies. Measurement of biomarkers of chronic stress could have given a more comprehensive view of the stress that lame sows experienced, as it has been reported in cows^21^.

Moderate lameness and severe lameness during pregnancy did not affect litter performance data before 27 days of age. In the case of prolificacy, lameness has not been reported as a relevant factor that alters reproductive variables such as the number of live piglets^47^. However, individual piglet weight at 27 days of age was different between groups, being significantly lower in piglets G3 compared to G2 and showing a similar tendency in comparisons with G1. This result could have several explanations from the standpoint of nutritional, behavioral, hormonal, or metabolic mechanisms. When nutrition is not ideal during pregnancy, the metabolism of the fetus can be altered during the neonatal period. In our experiment, sows were fed individually during pregnancy, which meant that no effect of lameness on the ability of the sow to compete for food could account for the lower piglet body weight at weaning^25^. Feeding regime during lactation was the same for all the sows, but consumption was not controlled so if lame sows consumed a lesser quantity of food, it could, potentially affect milk production^48,49^. The aspect of a possible difference in food consumption, together with increased lying behavior reported in lactating lame sows^7^, is a plausible alternative explanation for the reduced piglet weight at 27 days of age, in the offspring of lame sows. This does not involve the in-utero experience. Shoulder ulceration is common in lactating sows housed in farrowing stalls producing decrease in nursing frequency^50^. In our data, none of the sows showed shoulder ulcers, however we did not collect data on frequency of nursing. It is important to mention that the measures of lameness were collected during pregnancy and at the time of parturition they were resolved. Certainly, measures of nursing events, milk intake and milk quality would be important to characterize the nutritional impact of lameness on the offspring. Another explanation is that glucocorticoids or cytokines intervene in a catabolic way in growth processes^51^, and in previous studies, where individuals have been treated with glucocorticoids during pregnancy, it was found that their offspring had lower weight at birth^52^. In our study, we did not find evidence of a higher concentration of glucocorticoids generated by stress or pain in lame sows. In addition the proper functioning of the enzyme 11β-HSD-2 might be also affected, allowing a greater passage of glucocorticoids to the fetus without prior inactivation^43,53^. We did not measure glucocorticoids in placental tissue during pregnancy^54^, and our data on the relationship between placental cortisol and cortisone at farrowing suggest that the placenta was efficient in inactivating cortisol to cortisone, but it is important to mention that sows where moved from group housing to individual farrowing pens and as result the placental concentration of cortisol and cortisone may not reflect the events when they did show lameness. Other possibilities are that the metabolic costs associated with coping with lameness that could have compromised the offspring during the prenatal and early postnatal period. In humans, low weight at birth is associated with morbidity in adulthood^55^, which leaves us with an open research window to conduct experiments that monitor morbidity in animals born from females with a high or low degree of prenatal stress, especially lameness. The placental concentration of cytokines, which are released in response to inflammatory processes, during pregnancy appears to be higher in smaller piglets when compared with large animals^56^. More studies should be conducted to elucidate the causal mechanisms that may affect the performance of piglets.

As proposed in our hypothesis, a lower number of skin lesions was recorded in piglets from G1 than G2 on days 28 and 29 of age. This result indicates that piglets born from sows without lameness cope better with challenging social situations, probably avoiding agonistic interactions, when compared with the offspring from sows with moderate lameness. No differences were found relating to skin lesions involving piglets from sows with severe lameness, most likely due to the pain relief offered to this group of sows which probably attenuated the effects of severe lameness. Piglet aggression has been associated with compromised memory processes, resulting from the disruption of stress-responsive genes in prematurely weaned pigs^57,58^. We did not measure whether memory processes varied between G1, G2, and G3, piglets, but this would be interesting to explore.

Vocalization was more frequent in piglets from G1 for both open field and novel object tests. These tests are recognized as fear tests, because they impose on the animals a novel and open area, in social isolation, and also a novel object, so that conflicting motivations such as avoidance and exploratory behavior can be measured^59^. Vocalizations, according to their characteristics, are considered as an indicator of negative or positive emotions in different species, including domestic pigs^60–63^. In our results, the emotional valence is difficult to determine since we do not know the characteristics of the vocalizations. Vocalization in piglets could have a beneficial evolutionary role when exposed to social isolation or situations that represent a negative emotional valence^64,65^. Nevertheless, it would be worth comparing vocalizations in piglets exposed to fear tests in other contexts to make a better comparison, also adding analysis of the acoustic characteristics of these vocalizations, to better analyze associated emotions.

To our knowledge, this is the first study investigating the effects of lameness in sows during gestation on developmental outcomes in the offspring. Here we demonstrated that lameness in pregnant sows, especially moderate lameness, has negative effects on the offspring affecting weight gain and increasing number of skin lesions. We also demonstrated altered reactivity during fear tests, indicated by a decreased vocalization in piglets from sows with moderate lameness. Additionally, since there were no differences in cortisol concentration in saliva or placenta tissue, we suggest that other mechanisms, such as cytokines or epigenetics markers, may be involved in the phenotypic outcomes that we demonstrated, and this needs further investigation. Finally, it is worth emphasizing the consequences of ethical concerns to reduce the pain and suffering in lame animals in our care, especially in contexts where effects may pass to the next generation, particularly when we have the knowledge to assess and mitigate this condition in sows.

## Supplementary Information

Supplementary Information.

## Data Availability

The datasets generated and analyzed during the current study are available from the corresponding author on reasonable request.
