# Supplementary material for: The in-utero experience of piglets born from sows with lameness shapes their life trajectory
Source: Sci Rep. 2021 Jun 22;11:13052. doi: 10.1038/s41598-021-92507-2 (PMC8219680; doi:10.1038/s41598-021-92507-2)
Supplement: Supplementary file 1 — Supplementary Information. [file 41598_2021_92507_MOESM1_ESM.docx]

**The *in-utero* experience of piglets born from sows with lameness shapes their life trajectory**

Marisol Parada Sarmiento^1,2^* Thiago Bernardino^1^; Patricia Tatemoto^1^; Gina Polo^3^; Adroaldo José Zanella^1^*.

^1^ Center for Comparative Studies in Sustainability, Animal Health and Welfare, Department of Preventive Veterinary Medicine and Animal Health, School of Veterinary Medicine and Animal Science, University of São Paulo, Campus Ferando Costa, Av. Duque de Caxias Norte, 225 Caixa Postal 23, CEP 13635-900, Pirassununga, SP-Brazil;

^2^ Faculty of Veterinary Medicine, University of Teramo, Piano d’Accio 64100, Teramo, Italy;

^3^ Grupo de Investigación en Epidemiología y Salud Pública. Universidad de La Salle. Bogotá, Colombia.

* mparadasarmiento@unite.it; * adroaldo.zanella@usp.br

**Supplementary Figure S1. Experimental design.** Timeline of the experiment, from insemination in the sows to the fear tests in the offspring.

To identify the possible impact of the sow diet in the experiment, a multiple correspondence analysis was carried out and no effects were found between the previous experiment and our current findings. Based in the supplementary Fig. S2 - 1, it is possible to observe an association between the high fiber diet of the mothers and belonging to the group G1. Additionally, with fewer skin lesions at days 29 and 30, birth weight between 1.7-1.88 Kg, weight at day 21 between 4.2-5.8 kg and weight at day 27 between 5.6 -7.95 Kg. Likewise, feeding of sows with low fiber diets is associated with belonging to the G2, with heavier piglets at birth between 1.88-2.5 Kg and at weeks 21 (7-9.1 Kg) and 27 (8.8-13 Kg), greater number of skin lesions on days 28, 29 and 30 and more vocalizations in the novel object test. However, when the relationship is observed at the level of individuals (see supplementary Fig. S2 - 2 for details) a clear differentiation is not observed between the piglets of sows fed with high and low fiber, being noticeable an important overlap.


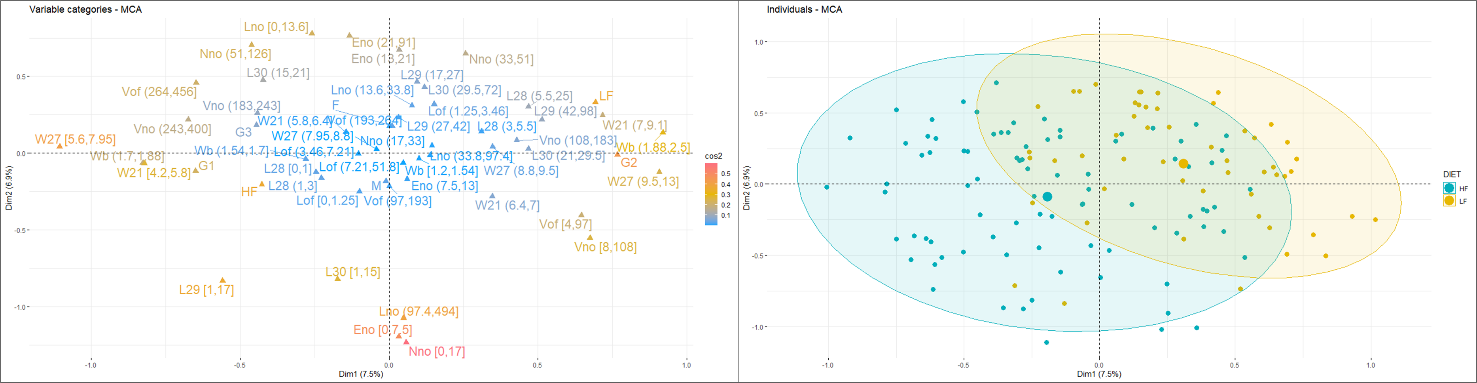


❶

❷

**Supplementary Figure S2.** Graphic result of the multiple correspondence analysis to show squared correlations between variables of piglets and the dimensions are used as coordinates identified. The description of each variables abbreviation in the graph **(1)** follows: high fiber (HF) and low fiber (LF) diet; piglets from sows without lameness (G1), from sows with moderate lameness (G2) and from sows with severe lameness (G3); weight at birth (Wb), at 21 (W21) and at 27 (W27) days of age; skin lesions at 28 (L28), 29 (L29) and 30 (L30) days of age; latency (Lof) and vocalizations (Vof) in the open field test; latency (Lno), exploration (Eno), near to the object (Nno) and vocalizations (Vno) in the novel object test. The graph **(2)** shows the relationship between piglet’s variables – at the level of individuals, and sow dietary treatments – high fiber diet (HF) and low fiber diet (LF).

According to Supplementary Figure S3 - 1 , in which the squared correlations between sow variables are identified, it is possible to observe an association between feeding with low fiber and some variables such as number of total born piglets and live born piglets, cortisol ratio in the morning and late on days 75 and 90, and average weight on days 21 and 28. However, when the relationship is observed at the individual level (Supplementary Figure S3 - 2) there is no association between the type of diet and the group according to the degree of lameness.


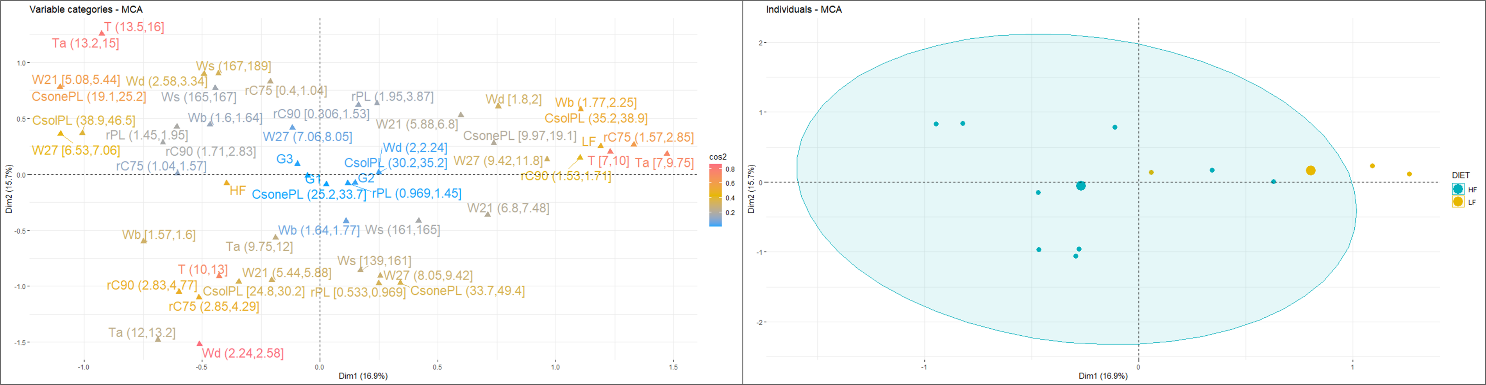


❶

❷

**Supplementary Figure S3.** Graphic result of the multiple correspondence analysis to shows squared correlations between sow variables are identified. The description of each variable’s abbreviation in the graph **(1)** follows: total born piglets (T) and total alive born piglets (Ta); average weight at birth (Wb), at 21 (W21) and at 27 days (W27) of age; average daily weight gain (Wd); sow weight (Ws); salivary cortisol ratio (morning and afternoon concentrations) at 75 (rC75) and 90 (rC90) days of pregnancy; placental cortisone (CsonePL) and cortisol (CsolPL) concentration; and ratio between placental cortisone and cortisol concentration (rPL). The graph **(2)** shows the relationship between sow’s variables – at the level of individuals, and sow dietary treatments – high fiber diet (HF) and low fiber diet (LF).

| **Sows groups** | **Day** | **Time** | **N** | **Mean (pg/50µl)** | **s.d.** | **Mean ratio** |
| --- | --- | --- | --- | --- | --- | --- |
| **G1** | 75 | 06:00 | 7 | 0.414 | 0.285 | 2.076 |
|  |  | 18:00 | 6 | 0.243 | 0.124 |  |
|  | 90 | 06:00 | 7 | 0.510 | 0.312 | 2.871 |
|  |  | 18:00 |  | 0.213 | 0.089 |  |
| **G2** | 75 | 06:00 | 9 | 0.331 | 0.255 | 2.826 |
|  |  | 18:00 |  | 0.190 | 0.102 |  |
|  | 90 | 06:00 | 10 | 0.472 | 0.226 | 2.452 |
|  |  | 18:00 |  | 0.204 | 0.055 |  |
| **G3** | 75 | 06:00 | 4 | 0.240 | 0.102 | 1.362 |
|  |  | 18:00 | 5 | 0.204 | 0.072 |  |
|  | 90 | 06:00 | 4 | 0.554 | 0.248 | 2.381 |
|  |  | 18:00 | 5 | 0.654 | 1.078 |  |

**Supplementary Table S1:** Descriptive measures from saliva cortisol collected twice daily (at 06:00 and 18:00) from sows at 75 and 90 days of pregnancy. Number of animals is abbreviated as N. Standard deviation is abbreviate as s.d. Average of each variable is presented as *mean*.

| **Sows groups** | **Placental hormone** | **N** | **Mean** | **s.d.** | **p-value of t-test** |
| --- | --- | --- | --- | --- | --- |
| **G1** | Cortisol | 6 | 3.537 | 0.684 | 0.024 |
|  | Cortisone |  | 22.954 | 14.842 |  |
| **G2** | Cortisol | 8 | 3.900 | 0.570 | 0.004 |
|  | Cortisone |  | 26.537 | 15.183 |  |
| **G3** | Cortisol | 5 | 3.983 | 1.489 | 0.032 |
|  | Cortisone |  | 16.489 | 8.785 |  |

**Supplementary Table S2:** Descriptive measures of placental cortisol/cortisone concentrations and results of intraspecific comparisons in the sows groups G1, G2 and G3.

| **Measure** | **Sow group G1 (N=7)** | | **Sow group G2 (N=10)** | | **Sow group G3 (N=5)** | |
| --- | --- | --- | --- | --- | --- | --- |
|  | Mean | s.d. | Mean | s.d. | Mean | s.d. |
| Average daily litter weight gain (Kg) | 2.295 | 0.248 | 2.402 | 0.501 | 2.647 | 0.538 |
| Average daily weight gain per animal (Kg) | 0.224 | 0.047 | 0.238 | 0.043 | 0.242 | 0.036 |
| Gestation length (days) | 115 | 0.577 | 114 | 1.886 | 114.2 | 1.095 |
| Number of crushed piglets | 0.571 | 0.787 | 1.3 | 1.059 | 0.6 | 0.548 |
| Total number of piglets born | 11.428 | 2.878 | 12.8 | 2.300 | 12.8 | 2.168 |
| Total number of piglets born alive at farrowing | 11.143 | 2.609 | 11.9 | 2.331 | 12 | 1.871 |
| Total litter weight at 21 days of age (Kg) | 65.6 | 9.958 | 65.24 | 16.010 | 67.76 | 7.516 |
| Total litter weight at 27 days of age (Kg) | 83.514 | 8.206 | 87.86 | 16.461 | 90.56 | 17.032 |
| Total litter weight at farrowing (Kg) | 17.06 | 2.459 | 18.827 | 2.690 | 19.35 | 2.505 |

**Supplementary Table S3:** Descriptive measures of performance data from the sow groups G1, G2 and G3.

| **Measure** | **Age (days)** | **Piglets group G1** | | | **Piglets group G2** | | | **Piglets group G3** | | | |
| --- | --- | --- | --- | --- | --- | --- | --- | --- | --- | --- | --- |
|  |  | N | Mean | s.d. | N | Mean | s.d. | N | Mean | s.d. |  |
| Weight (Kg) | 0 | 74 | 1.61 | 0.24 | 113 | 1.67 | 0.36 | 57 | 1.70 | 0.25 |  |
|  | 21 |  | 6.21 | 1.28 | 105 | 6.21 | 1.14 |  | 5.94 | 1.12 |  |
|  | 27 | 73 | 8.00 | 1.54 | 104 | 8.45 | 1.86 |  | 7.95 | 1.68 |  |
| Number of skin lesions | 28 | 52 | 2.69 | 2.55 | 66 | 5.08 | 4.65 | 38 | 2.95 | 3.59 |  |
|  | 29 |  | 24.79 | 18.03 |  | 32.49 | 16.10 |  | 30.58 | 19.96 |  |
|  | 30 |  | 22.42 | 13.02 |  | 23.45 | 11.84 |  | 23.24 | 14.05 |  |

**Supplementary Table S4:** Descriptive measures of weight at birth at 21, and 27 days of age; and skin lesions at 28, 29 and 30 days of age in piglets from groups G1, G2 and G3.

| **Measure** | **Test** | **Piglets group G1 (N=47)** | | **Piglets group G2 (N=62)** | | **Piglets group G3 (N=33)** | |
| --- | --- | --- | --- | --- | --- | --- | --- |
|  |  | Mean | s.d. | Mean | s.d. | Mean | s.d. |
| Latency● | Open field | 6.53 | 10.380 | 5.658 | 6.993 | 5.484 | 6.050 |
| Central Quadrants● |  | 29.979 | 10.109 | 29.871 | 12.609 | 26.909 | 7.341 |
| Lateral Quadrants● |  | 56.915 | 24.437 | 60.193 | 30.040 | 57.515 | 18.762 |
| Activity● |  | 87.319 | 30.029 | 89.097 | 38.451 | 84.667 | 24.729 |
| Vocalizations▲ |  | 219.126 | 98.028 | 170.790 | 102.573 | 183 | 99.936 |
| Latency● | Novel object* | 83.614 | 108.047 | 71.659 | 84.043 | 60.473 | 66.511 |
| Exploration● |  | 17.191 | 14.979 | 14.164 | 9.510 | 18.667 | 18.261 |
| Near to the object● |  | 41.532 | 28.993 | 31.836 | 21.143 | 37.818 | 27.617 |
| Vocalizations▲ |  | 221.510 | 97.801 | 160.541 | 87.347 | 177.848 | 81.221 |

**Supplementary Table S5:** Descriptive measures from the variables analyzed during open field and novel object test. ● The unit of these measures is seconds. ▲ The unit of these measures is in frequency; it means the number of all types of vocalizations realized during 300 seconds for each test. *The number of individuals in the group G2 were 61.
